# Supplementary material for: Further Delineation of Duplications of ARX Locus Detected in Male Patients with Varying Degrees of Intellectual Disability
Source: Int J Mol Sci. 2022 Mar 13;23(6):3084. doi: 10.3390/ijms23063084 (PMC8955779; doi:10.3390/ijms23063084)
Supplement: Supplementary file 1 [file ijms-23-03084-s001.zip › Supplementary Figure S1.pdf]

A

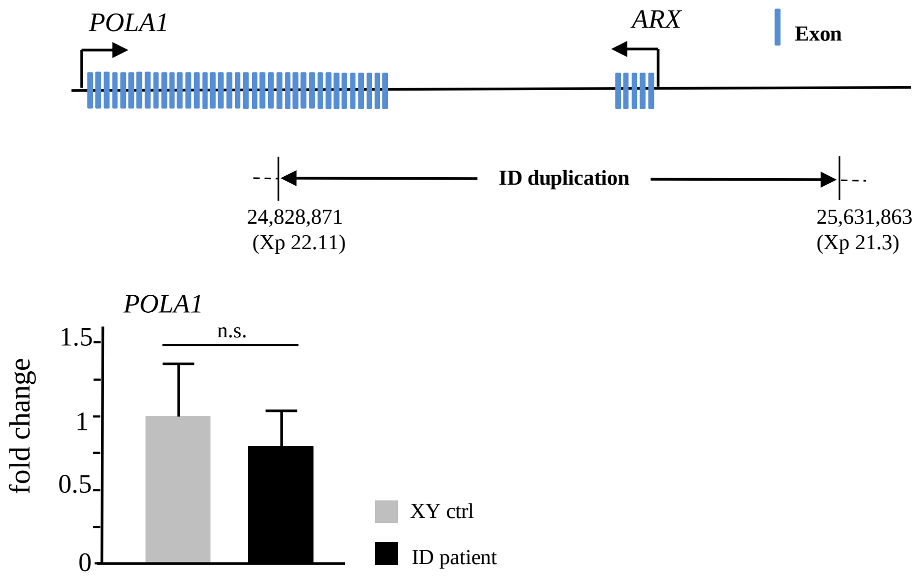

B

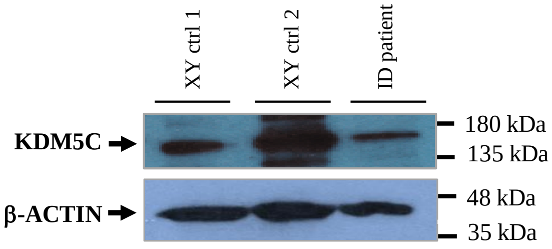

Figure S1. *POLA1* expression levels by RT-PCR study in lymphoblastoid cell lines of GE#1 ID patient and XYWT control (**A**). Results were normalized using *HPRT1* as reference gene and shown as mean $\pm$ SD from three independent experiments. A cartoon map of *POLA1* and *ARX* loci is shown.

Detection of KDM5C levels by Western blotting analysis in lymphoblastoid cell lines of GE#1 ID patient and XYWT controls. The western blotting experiments were repeated three times. As loading control, β-ACTIN was used. (**B**).
