# Supplementary material for: Further Delineation of Duplications of ARX Locus Detected in Male Patients with Varying Degrees of Intellectual Disability
Source: Int J Mol Sci. 2022 Mar 13;23(6):3084. doi: 10.3390/ijms23063084 (PMC8955779; doi:10.3390/ijms23063084)
Supplement: Supplementary file 1 [file ijms-23-03084-s001.zip › Supplementary materials. docx.pdf]

Supplementary Materials

Case description of the ID patient (#GE_1). Our proband is an 8-year-old boy, the child of healthy, non-consanguineous parents. Family history was unremarkable for neurodevelopmental disorders. He was born full-term by caesarean delivery with no perinatal issues and his growth parameters were adequate for gestational age. By the first year of life he presented with axial hypotonia and global neurodevelopmental delay. He was referred to us at the age of 8 years old for moderate intellectual disability, behavioural problems and dysmorphic features. His weight was 32 Kg (75th-90th centile), his height was 125 cm (25-50th centile) and his head circumference was 51 cm (25- 50th centile, falling into the full 50th centile at the 11 years old evaluation). On physical examination, he showed narrow forehead, convergent strabismus, large and fleshy ears, M shaped upper lip and undescended right testicle. During the first year of life, due to his history of hypotonia, he was studied by EEG that showed a global slight alteration of back ground rhythm and by EMG that resulted within the normal range. Both heart and abdominal ultrasounds were normal, except for a mild hepatosplenomegaly revealed by the early infancy. The legal guardian of the ID patient gave written informed consent for participation in the study.

Array-CGH analysis. Genomic DNA of the ID patient and his mother were extracted from peripheral blood lymphocytes using King Fisher Blood DNA Kit (Thermo Scientific, Waltham, MA, USA) according to manufacturers' instructions. Proband, his mother and reference DNA (Promega Corporation, Madison, WI, USA) were labelled with Cy5-dUTP and Cy3-dUTP respectively. Whole genome array-CGH were performed using Human Genome CGH Microarray Kit 8x60K (Agilent Technologies, Santa Clara, CA, USA) with an average resolution of 100 kb (Build37: Feb 2009-hg19) according to manufacturers' instructions. Images of the array were acquired with Agilent scanner G2505B and analysed with Feature Extraction software v9.5.1 (Agilent Technologies, Santa Clara, CA, USA). Graphical overviews of results were obtained with Genomic Workbench Standard Edition software v5.0.14 (Agilent Technologies, Santa Clara, CA, USA).

Quantitative Real-time PCR. Total RNA was isolated according to the TRIzol protocol (Life Technologies, Waltham, MA, USA). Reverse transcription was performed with QuantiTect Reverse Transcription kit (Qiagen, Hilden, Germany) and the steady-state mRNA abundance was determined using the Power SYBR Green PCR Master Mix (Applied Biosystems, Foster City, CA, USA) on the 7900HT Fast Real Time PCR System (Applied Biosystems, Foster City, CA, USA), as a standard procedure. Relative quantification of each gene was calculated using the ΔΔCt method and all data were normalized to HPRT as control gene. The oligonucleotide sequences for the human transcript analysis are here reported: F5'-TCCGACACCCAGCTTTCATC-3' and R5'-AGGATGTTGAGCTGCGTGAG-3' for *ARX*; F5'-CTCCTTGCTACGCTCCCACTACGA-3' and R5'-TCAAATGGACGTGTGTTACAC-3' for *KDM5C*; F5'-TGGGTTTTAGGACCAGGATG-3' and R5-GGTGCTGAAGCTGGCAGT-3' for *SYN1*; F5'-TCGGATGGGAATTATGTCACC-3' and R5'-TGTCCCGGCTTTGATCAGAA-3' for *POLA1*; F5'-AAGCAAAATACAAAGCCTAA-3' and R5'-ATTTTACTGGCGATGTCA-3' for *HPRT*. Each experiment assays were performed in triplicate in three independent experiments.

Immunoblotting and antibodies. Lymphoblastoid cells were collected by centrifugation and lysed in RIPA lysis buffer (50 mM Tris-HCl 7.5, 150 mM NaCl, 1 mM EDTA, 1% Triton-X 100) supplemented with 1X protease inhibitors cocktail (Roche, Basilea, Switzerland). Protein concentrations were measured using Biorad protein assay (Bio-Rad Laboratories, Hercules, USA). Lysates were added with Laemmli buffer and denaturated by heat. Proteins were loaded to an 8% sodium dodecylsulfate polyacrylamide gel (SDS-PAGE) and transferred to a nitrocellulose membrane (Merck Millipore, Burlington, USA). Membrane was blocked with 5% non-fat milk, and after incubated with human anti-KDM5C antibody diluted to 1:250 (Abcam, Cambridge, UK) and anti β-actin antibody diluted to 1:3000 (Santa Cruz Biotechnology, Heidelberg, Germany), used as a loading control. Secondary anti-mouse (Santa Cruz Biotechnology, Heidelberg, Germany) or anti-rabbit (Santa Cruz Biotechnology, Heidelberg, Germany) IgG HRP antibodies were used for

detection and diluted to 1:10.000. The signals were detected with an enhanced chemiluminescence kit (Advansta, San Jose, USA).

Cell lines, Luciferase reporter assays and plasmid constructs. HeLa, SH-SY5Y and HEK293T cell lines were maintained in Dulbecco's modified Eagle's medium (Life Technologies, Scotland, Swindon, UK) supplemented with 10% fetal bovine serum (FBS Life Technologies, Scotland, Swindon, UK), 100 units/ml penicillin and 100 mg/ml streptomycin. Patient lymphoblastoid cell line (LCLs) was established by Epstein-Barr virus transformation by Galliera Genetic Bank. LCLs were cultured in RPMI 1640 medium (Life Technologies, Scotland, Swindon, UK) supplemented with 20% fetal bovine serum (FBS, Life Technologies, Scotland, UK) and 1% antibiotics at 37° C in 5% CO2. Cell transient transfections were performed according to standard methods. The reporter activities were measured using the Dual-Luciferase Reporter Assay System (Promega, Madison, USA) as elsewhere reported [9,14]. Each assay was performed in duplicate in three independent experiments and the resulting firefly luciferase values were normalized using the Renilla values. All cell lines were tested regularly for mycoplasma and confirmed negative.

Polymer Physics 3D reconstruction. In the Strings and Binders Switch (SBS) polymer model [41], a chromatin filament is modelled as a string of beads, where binding sites are present and can interact with binders. To determine the number of binding sites and their position along the polymer chain, we used the PRISMR algorithm [27], a Machine Learning computational approach that takes in input the experimental HiC map and extracts the sequence of binding domains that best describes the architectural data, based uniquely on Polymer Physics laws. Finally, Molecular Dynamics simulations were performed using the parameters described in [42] with the free software LAMMPS (Plimpton 1995).

Statistical and bioinformatics analysis. The statistical analyses were performed with the GraphPad Prism 4 software (GraphPad Software) to calculate the significance of the differences among each sample against the control. The student's t-test was used. Extensive use was made of the UCSC genome browser and several public available databases including the Database of

Genomic Variants, DECIPHER, (https://www.deciphergenomics.org/), VISTA Enhancer Browser (https://enhancer.lbl.gov/) and a Database for human and mouse development gene expression (http://www.humous.org/). All genomic coordinates correspond to the human genome assembly build 36 (hg38).
